# Deep learning in cancer pathology: a new generation of clinical biomarkers

**DOI:** 10.1038/s41416-020-01122-x

**Published:** 2020-11-18

**Authors:** Amelie Echle, Niklas Timon Rindtorff, Titus Josef Brinker, Tom Luedde, Alexander Thomas Pearson, Jakob Nikolas Kather

**Affiliations:** 1 Department of Medicine III, University Hospital RWTH Aachen, Aachen, Germany; 2 German Cancer Research Center (DKFZ), Heidelberg, Germany; 3 National Center for Tumor Diseases (NCT), German Cancer Research Center (DKFZ), Heidelberg, Germany; 4 Department of Gastroenterology, Hepatology and Infectious Diseases, University Hospital Duesseldorf, Düsseldorf, Germany; 5 Section of Hematology/Oncology, Department of Medicine, The University of Chicago, Chicago, IL USA

**Keywords:** Targeted therapies, Tumour biomarkers, Computational science, Cancer imaging

## Abstract

Clinical workflows in oncology rely on predictive and prognostic molecular biomarkers. However, the growing number of these complex biomarkers tends to increase the cost and time for decision-making in routine daily oncology practice; furthermore, biomarkers often require tumour tissue on top of routine diagnostic material. Nevertheless, routinely available tumour tissue contains an abundance of clinically relevant information that is currently not fully exploited. Advances in deep learning (DL), an artificial intelligence (AI) technology, have enabled the extraction of previously hidden information directly from routine histology images of cancer, providing potentially clinically useful information. Here, we outline emerging concepts of how DL can extract biomarkers directly from histology images and summarise studies of basic and advanced image analysis for cancer histology. Basic image analysis tasks include detection, grading and subtyping of tumour tissue in histology images; they are aimed at automating pathology workflows and consequently do not immediately translate into clinical decisions. Exceeding such basic approaches, DL has also been used for advanced image analysis tasks, which have the potential of directly affecting clinical decision-making processes. These advanced approaches include inference of molecular features, prediction of survival and end-to-end prediction of therapy response. Predictions made by such DL systems could simplify and enrich clinical decision-making, but require rigorous external validation in clinical settings.

## Background

Decision-making processes in oncology today no longer rely on workflows that are linear and straightforward; rather, with the availability of an ever-increasing number of biomarkers, these flowcharts resemble intricate trees with numerous branches, which consequently increase the complexity of treatment recommendations for solid tumours. Currently used molecular biomarkers in these oncology workflows can be prognostic or predictive. Prognostic biomarkers allow the categorisation of patients according to their risk of disease progression or death and, accordingly, can be used to adjust treatment intensity for individual patients. For example, in stage II colorectal cancer (CRC), microsatellite instability (MSI) is a prognostic biomarker; if MSI is detected, a lower treatment intensity of adjuvant chemotherapy can be used due to the inherently better prognosis of these patients.^1^ By contrast, predictive biomarkers enable a particular targeted treatment to be chosen for a specific patient group. For example, in treatment-refractory stage IV CRC, MSI is an FDA-approved biomarker for immune-checkpoint-inhibitor-based immunotherapy.^2^ In this case, the detection of MSI correlates with the likelihood of a positive therapeutic response, making MSI a strong predictive biomarker in this setting. Similarly, in breast cancer, the detection of *HER2* positivity^3^ makes patients eligible for treatment with anti-HER2 agents, thus acting as a strong predictive biomarker in this disease.^4^ The choice of treatment for non-small-cell lung cancer (NSCLC) is influenced by a high number of molecular biomarkers,^5^ with oncogenic mutations in the gene encoding epidermal growth factor receptor (EGFR) and other genes, gene fusions of anaplastic lymphoma kinase (ALK) or other drivers and the overexpression of programmed cell death ligand 1 (PD-L1)^6^ being part of the standard-of-care molecular panel required for routine treatment of advanced or metastatic disease.^7^ It is clear, then, that the rapidly increasing number and clinical importance of molecular biomarkers in routine clinical practice allows cancer treatments to be tailored more specifically according to the genetic make-up of a particular tumour; consequently, however, the cost, turnaround time and tissue requirements in routine workflows also increase.^8,9^

The design of clinical trials for new therapeutic agents in solid tumours is increasingly coupled to predictive biomarkers. In addition to highly prevalent molecular features, many Phase 2 and 3 trials carried out over the past ~5 years have focused on rare molecular subpopulations of solid tumours, such as those with MSI,^10^ homologous repair deficiency^11^ and fusion-driven tumours across cancer types.^12–14^ As mentioned above, MSI is used as a predictive biomarker for immunotherapy, while homologous repair deficiency tumours are effectively targetable by inhibitors of poly ADP-ribose polymerase (PARP), and fusion-driven tumours respond exceptionally well to molecularly targeted therapy. However, with the prevalence of these genotypes ranging between 1% and 10% in real-world populations, screening potential participants for these trials is costly and hampered by the limited availability of molecular assays. So, despite the increased number of prognostic and predictive biomarkers enabling a more nuanced treatment of cancer patients, the complexity of clinical decision-making processes increasingly becomes an issue in clinical routine and clinical trial recruitment.

Although most new biomarkers in oncology are based on molecular biology assays, advances in deep learning (DL) are facilitating the extraction of otherwise hidden information directly from routinely available data. DL is a method in the realm of artificial intelligence (AI) that makes use of artificial neural networks to identify recurring patterns in complex datasets. Image data in particular has a high information density, making it ideal for analysis with DL techniques.

**Fig. 1:**
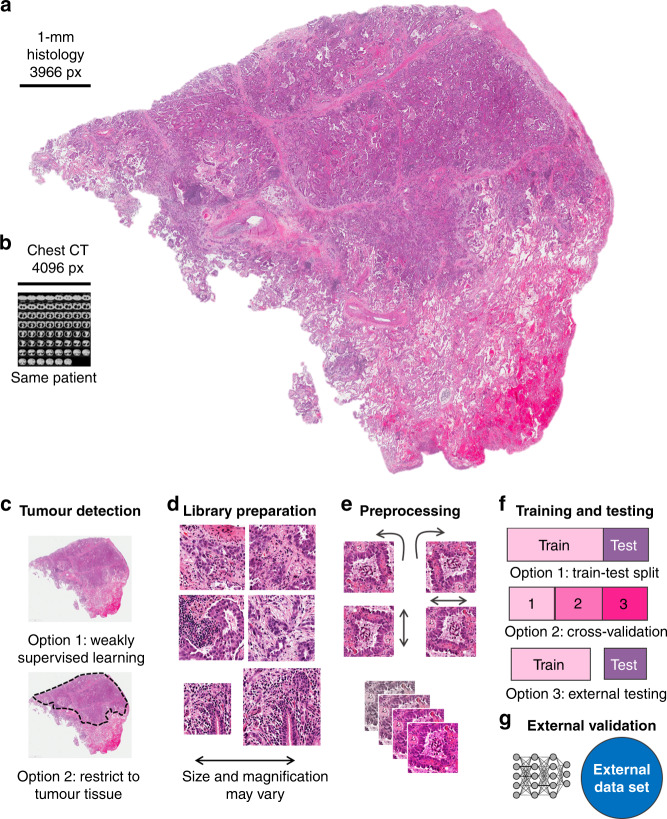
Consensus pipeline of deep learning in pathology. **a** Routine histology image of lung cancer (from The Cancer Genome Atlas (TCGA) and The Cancer Imaging Archive (TCIA)). **b** Size comparison (in terms of pixels) of a chest CT scan of the same patient. **c** Consensus image-processing pipeline. First, either the whole slide or just the tumour region is tessellated into smaller image tiles. **d** These tiles comprise an image library, similar to the library preparation (prep.) in genome sequencing. **e** Tiles are preprocessed to achieve rotational constancy and augment the dataset. **f** Deep- learning classifiers are developed and deployed by splitting the patient cohort into a training and testing set, by using cross-validation or by having multiple cohorts available for training and testing. **g** Ideally, an additional external dataset is used for validation of the resulting classifier.

Indeed, DL-based image analysis has broad applications in multiple fields of modern medicine that involve image data: in radiology, DL performs repetitive tasks with human-like, or super-human, performance, such as tumour detection or organ segmentation on computer tomography (CT) images. To date, more than a dozen DL methods are approved for clinical use in radiology by the FDA—for example, DL-based analysis of CT data was carried out in a 2019 lung cancer screening trial,^15^ and evidence on the clinical usefulness of these methods is quickly mounting. Magnetic resonance imaging (MRI) data, which contain much more information than CT data, are also amenable for DL-based mining,^16^ and DL has also shown robust results for non-radiology tasks such as the analysis of real-time endoscopy images^17,18^ and skin cancer detection in dermoscopy images.^19,20^ Compared to these imaging modalities, however, histology is a ubiquitous image source with a remarkable information density that can be derived from routine clinical practice. Being much larger than radiological images in terms of pixels, images from histology slides carry much more information: millions of different cells can be seen in a histology slide and their morphology and spatial arrangement carry much more information than other medical images. Even the size of a whole chest CT dataset does not get close to the size of the dataset from one histological whole- slide image derived from the tumour of the same patient when measured in pixels (Fig. 1a, b). This high information density makes histological images an attractive source for DL-based biomarker extraction.

With this paper, we aim to enable a clear overview of DL applications in the field of cancer histology by the categorisation and comparison of DL-based studies from a clinical point of view. Subsequently, possible use cases and necessary further steps on the way to beneficial usage in a clinical setting will be discussed.

## Deep-learning-based analysis of histology images

During the diagnostic workup of patients with solid tumours, tissue samples are usually obtained either by biopsy or by surgical resection, followed by pathological preparation and in most cases staining by haematoxylin and eosin (H&E). Therefore, H&E slides are routinely available for almost every cancer patient, making them an easy-to-obtain, information-rich data source for the assessment by DL methods, also explaining the focus of previous studies on these types of images. Nevertheless, DL is a tool with applicability in different types of histological stains, such as immunohistochemistry (IHC)^21^ or periodic acid-Schiff.^22^

### Image-processing steps

The sheer data size of scanned whole-slide histology images poses practical challenges for the analysis of DL-based images. Their large file size does not enable them to be loaded entirely onto the memory of graphics-processing units (GPUs), the workhorse of DL. Furthermore, histology images usually contain an abundance of non-tumour tissue, which dilutes the overall information content. To deal with such large and heterogeneous images, extensive preprocessing of these images is required; consequently, a consensus image analysis pipeline has been created. This step-by-step analysis includes tessellation (Fig. 1d), preprocessing of image tiles (Fig. 1e) and training and testing of a DL network, also called DL classifier (Fig. 1f), that can then be applied to external validation cohorts (Fig. 1g). The term “classifier” refers to any computer programme that—after being trained on a set of examples—can subsequently categorise similar data. In histology image analysis, a classifier can categorise small image patches as “tumour” or “non-tumour”, or it can classify patients as “potential responders” or “potential non-responders”. Among all classifiers, DL networks are emerging as the most widely used and most powerful technology.

### Basic and advanced applications of DL in cancer histology

Following standardised preprocessing procedures, histological images can be used for a range of DL applications. DL workflows use a training cohort of patients to predict a predefined label from image data. Previous studies have explored a variety of labels, ranging from predicting the presence of invasive tumour tissue in prostate tissue,^23–25^ to determining tumour genotype directly from histology images.^26,27^ Here, we propose that these types of label are distinguished on the basis of their use in basic or advanced DL applications (Supplementary Fig. 1).

Basic DL applications aim to simplify routine workflows that are currently entirely performed by human pathologists. Prominent examples are the detection of tumour tissue in biopsy samples or tumour subtyping based on morphology, such as Gleason scoring of prostate cancer samples. In the latter case, the numerical value of the Gleason score is used as a label for training a DL system. These basic DL applications can potentially decrease cost and turnaround time in pathology departments, but do not change the ultimate readout upon which clinicians base their treatment recommendations.

Advanced DL applications, on the other hand, go beyond the standard reporting that is currently performed by pathologists. One example is the prediction of genetic mutations and survival directly from H&E-stained tissue slides. In the case of genetic mutations, the image label is the genotype as determined during the conventional diagnostic workup using a molecular biology assay or other gold standard tests as the ground-truth method. “Ground truth” refers to the type of assay used to label images during training. Thus, the DL classifier can be trained to reproduce the “ground truth” (also called “gold standard method”) just by analysing histology image data. Unlike basic DL applications, such advanced applications of DL can provide clinicians with additional information that is not being extracted from routine material in current clinical workflows: these applications constitute a new class of biomarkers with potential prognostic and/or predictive information (Supplementary Fig. 1).

DL is thus a powerful tool with which to extract information from histology images of solid tumours, and can be used to automate current workflows or to provide additional information that is currently not being used in clinical workflows. In the next few sections, we will summarise the current status of basic and advanced applications of DL in cancer histology image analysis.

## Basic applications of DL: tumour detection, grading and subtyping

In general, every sample of a solid tumour undergoes detailed analysis by a trained pathologist who confirms the presence of tumorous tissue and provides further information such as grade and subtype of the tumour sample at hand. In the field of those basic but important diagnostic tasks, DL has shown potential to be useful to automate repetitive tasks in diagnostic pathology.

### Automating histopathology workflows by DL

For many years, digital pathology publications have described and iteratively refined basic image analysis tasks such as tumour detection,^28^ tumour subtyping,^29^ quantification of cell numbers^30^ and classification of cell types.^31^ What these approaches have in common is that the ground-truth method and the DL system use the same image data as input for their prediction. For example, the presence of invasive tumour tissue in prostate cancer biopsy samples is normally assessed from H&E-stained tissue slides by a pathologist. A basic DL system recapitulates this task and is trained to predict the presence of invasive cancer from the same H&E histology image. Thus, such DL-based tumour detectors can automate tedious tasks that are normally performed manually.

**Fig. 2:**
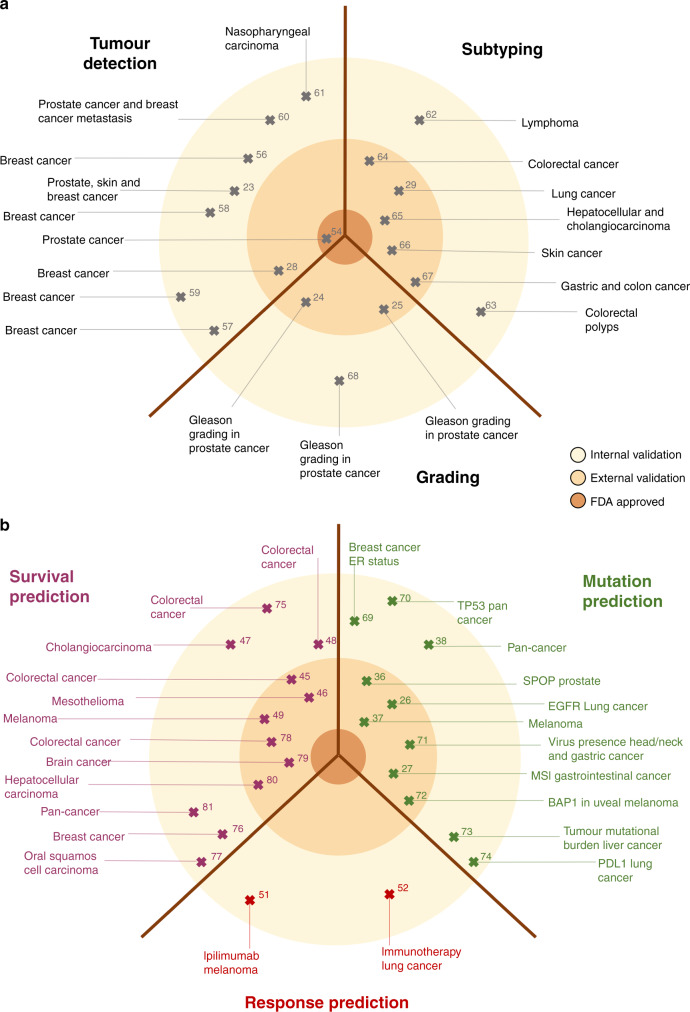
Clinical applications of basic and advanced deep-learning (DL) image analysis in histopathology. DL pathology can be applied to tumour detection and identification of subtype (basic applications) or to predict clinical features of interest (advanced application). Published studies (indicated by reference number) are classified according to the level of evidence (monocentric (internally approved), multicentric (externally approved) or FDA approved). **a** Basic image analysis tasks, including tumour detection, grading and subtyping. **b** Advanced image analysis tasks, including those that exceed pathologists’ routine capacities, such as prediction of mutation, prognosis and response. AI   artificial intelligence, NSCLC    non-small-cell lung cancer, WSI   whole-slide image, ER   oestrogen receptor, MSI   microsatellite instability, GI   gastrointestinal, SPOP   speckle-type BTB/POZ protein, BAP1   BRCA-associated protein 1, HNSCC   head and neck squamous cell carcinoma, CCA   cholangiocarcinoma.

Numerous studies, identified by a predefined search query on the MEDLINE database as shown in Supplementary Methods, have demonstrated the robustness of such DL-based tumour detection approaches across a range of tumour types, as summarised in Table 1 and Fig. 2a. Classification performance, meaning how well a DL classifier predicts a pre-specified endpoint, is typically measured by the area under the receiver-operating curve (AUROC), and DL-based tumour detectors often achieve AUROC values >0.99, indicating the almost complete accordance of the results from pathologists and DL networks. Other potential basic image analysis problems relate to recapitulating tumour detection and subtyping based on histological features. For example, the Gleason system is the single most relevant morphological biomarker used for patient stratification in prostate cancer. Gleason grading is usually performed manually by expert pathologists based on H&E tissue slides, but DL systems have been successfully applied to automate this task.^23^ Similarly, classifying NSCLC into adenocarcinoma or squamous cell carcinoma has clinical relevance and is reproducibly and quickly performed by expert pathologists and DL systems alike.^26^

**Table 1:** Comparison of basic DL image analysis studies in digital pathology, comprising tumour detection, subtyping and grading.

Reference	Description	Ext. validation	Number of slides	Number of patients	Number of cohorts	AUROC	*F* score	Accuracy	Other metrics
*Tumour detection*									
^28^	Detection of breast cancer tissue on whole-slide images	Yes	605	N/A	4	0.9	N/A	N/A	PPV = 0.72; NPV = 0.97; TPR = 0.87; FPR = 0.08; FNR = 0.13
^56^	Image-wise classification in four classes: normal tissue, benign lesion, in situ carcinoma and invasive carcinoma	No	258	N/A	1	N/A	N/A	77.8%	N/A
^57^	Classification of breast cancer tissue into benign versus in situ versus invasive versus normal	No	1495	N/A	2	N/A	N/A	85% (four classes)	N/A
^58^	Image-wise classification in four classes: normal tissue, benign lesion, in situ carcinoma and invasive carcinoma	No	400	N/A	1	0.97 (benign vs. malignant)	N/A	87.2% (four classes)	Sensitivity = 96.5% (benign vs. malignant); specificity = 88% (benign vs. malignant)
^23^	Detection of prostate carcinoma, basal cell carcinoma and breast cancer metastasis in axillary lymph nodes in the biggest cohort so far	No	44,715	15,187	3	0.99 (prostate); 0.99 (skin); 0.99 (breast metastasis)	N/A	N/A	N/A
^59^	Classification of breast cancer tissue into benign versus in situ versus invasive versus normal	No	2495	N/A	2	0.96–0.987	N/A	90%	N/A
^60^	Real-time processing of images by deep learning captured through the microscope in order to detect metastatic breast cancer in lymph nodes and to identify prostate cancer	No	66	N/A	3	0.97 (lymph-node metastasis); 0.99 (prostate)	N/A	N/A	N/A
^61^	Identification of malignant tissue in nasopharyngeal biopsies	No	726	726	1	0.99	N/A	N/A	N/A
*Tumour subtyping*									
^62^	Deep learning used for lymphoma subtyping	No	375	N/A	3	N/A	N/A	96.58%	N/A
^63^	Classification of five types of colorectal polyps	No	697	N/A	1	N/A	0.88	N/A	Sensitivity = 88.3%; PPV = 0.9
^64^	Consensus molecular subtyping of colorectal cancer	Yes	1553	1109	2	0.85	N/A	N/A	N/A
^29^	Classification of lung cancer into adeno-, squamous cell- and small-cell lung carcinoma, normal	Yes	143	N/A	3	0.86	N/A	N/A	N/A
^65^	Real-time assistant on classification of hepatocellular carcinoma and cholangiocarcinoma	Yes	150	150	2	N/A	N/A	84.2%	N/A
^66^	Classification of skin cancer WSIs in basaloid, squamous, melanocytic or other subtypes	Yes	18,607	N/A	4	0.88–0.96 (four classes)	N/A	78%	N/A
^67^	Classification of gastric and colon cancer biopsies and specimens into adenocarcinoma, adenoma or non-neoplastic tissue	Yes	10,186	N/A	4	0.98 (stomach adenocarcinoma); 0.96 (colon adenocarcinoma)	N/A	95.6% (stomach)	N/A
*Tumour grading*									
^68^	Gleason grading of prostate cancer	No	312	N/A	1	0.93	N/A	77.12%	N/A
^25^	Gleason grading in prostate cancer	Yes	9001	N/A	4	N/A	N/A	N/A	Cohens *κ* = 0.83
^24^	Gleason grading in prostate cancer	Yes	5758	1243	2	N/A	N/A	N/A	Cohens *κ* = 0.723

*N/A* not available.

For each study, the level of evidence (presence of external validation), the number (#) of tissue slides, patients and patient cohorts as well as quantitative performance metrics are given, including area under the receiver-operating curve, AUROC; F score; accuracy; positive predictive value, PPV; negative predictive value, NPV; true-positive rate, TPR; false-positive rate, FPR; false- negative rate, FNR; other metrics (sensitivity, specificity and others) if reported in the study. This table is related to Fig. 2a.

Ultimately, however, basic DL systems for tumour detection, grading and subtyping are of limited interest to clinicians as they do not change clinical practice in oncology. Compared to expert pathologists, such systems could potentially reduce cost or turnaround time, but only in rare cases, do they improve sensitivity and specificity as compared to human expert observers. Thus, although basic DL systems can bring about profound changes in the way pathology is practiced, from an oncologist’s point of view, they do not immediately change clinical workflows and treatment recommendations for cancer patients.

### Clinical-grade validation of basic DL methods

The most challenging part of developing basic DL systems in digital pathology is their clinical validation. The use of only a single dataset for method development and validation carries the risk of overfitting, resulting in the creation of a DL system that performs well in that particular patient cohort, but does not generalise to external cohorts. Correspondingly, validation of the DL system in external datasets, ideally multicentre datasets, is paramount for its ultimate routine use and regulatory approval. The past 2 years have seen an increasing number of large-scale, multicentre studies of basic DL systems. For example, three independent studies have demonstrated DL systems for prostate cancer detection and grading with pathologist-level performance and external validation in large patient cohorts.^23–25^ A key point to take away from these large-scale efforts is that the performance of DL systems increases with patient number in the training set, reaching a plateau in performance after training on 10,000–15,000 histological whole-slide images,^23^ which indicates the need for tremendous amount of images and data when aiming for the development of sufficiently performing DL systems. These efforts mirror large-scale international studies using other imaging modalities, such as mammography imaging.^32^ Therefore, for simple image recognition tasks, DL systems could conceivably alleviate workload for human experts in the near future.

## Advanced applications: prediction of mutations, survival and response from histology

### Moving beyond basic applications of DL in histology image analysis

DL systems can approach human performance in tumour detection, grading and subtyping, but histology images contain an abundance of information that is currently not systematically exploited to guide treatment decisions in oncology. As we will discuss in the following sections, this abundance of information has been demonstrated by a number of studies that have used DL to infer high-level labels directly from H&E images. These high-level labels cannot be reliably inferred by human experts observing histology images, but require other methods in addition to routine histopathology. In particular, there is an increasing focus on predicting clinically relevant labels directly from histology in three major areas: inference of genetic alterations, prediction of survival and prediction of treatment response (Table 2 and Fig. 2b). Similar to research in the broader field of digital pathology, research in these three key applications of DL has been rapidly growing in the past few years (Supplementary Fig. 2a, b). Unlike basic image analysis techniques, these advanced applications of DL to histology image analysis have the potential to directly change clinical decision-making in the management of solid tumours. Here, we review the current state of clinically applicable DL pathology and its implications on clinical workflows as well as clinical trial design and recruitment.

**Table 2:** Comparison of advanced DL image analysis studies in digital pathology, comprising mutation prediction, prognostication and response prediction.

Reference	Description	Ext. validation	Number of slides	Number of patients	Number of cohorts	AUROC	*F* score	Accuracy	Other metrics
*Mutation detection*									
^69^	Prediction of ER status in breast cancer	No	859	859	1	N/A	N/A	84%	Sensitivity = 88%; specificity = 76%
^36^	Prediction of SPOP mutation in prostate cancer	Yes	365	N/A	2	0.86 (*P* = 0.0038)	N/A	N/A	N/A
^26^	Prediction of different genes in lung cancer and ext. validation of EGFR mutation	Yes	1975	N/A	3	0.68	N/A	N/A	N/A
^37^	Prediction of BRAF and NRAS in melanoma	Yes	361	N/A	2	0.75 (BRAF); 0.77 (NRAS)	N/A	N/A	N/A
^70^	TP53 mutation prediction	No	27,815	N/A	28	0.8 (stomach)	N/A	N/A	N/A
^71^	Detection of HPV in head and neck cancer; detection of EBV in gastric cancer	Yes	1031	1031	4	0.7 (HPV); 0.81 (EBV)	N/A	N/A	N/A
^27^	Prediction of microsatellite instability in colorectal, gastric and endometrial cancer	Yes	2108	1952	5	0.84 (CRC)	N/A	N/A	N/A
^38^	Pan-cancer prediction of gene expression	No	10,514	8725	28	0.81 (MSI)	N/A	N/A	N/A
^72^	Prediction of BAP1 expression in uveal melanoma	Yes	47	47	2	N/A	0.93	92.8%	Sensitivity = 92.1%; specificity = 91.1%
^73^	Prediction of tumour mutational burden in liver cancer	No	368	350	1	0.95	N/A	94.86%	N/A
^74^	Prediction of PD-L1 status in non-small- cell lung cancer patients	No	130	130	N/A	0.8 (*P* < 0.01)	N/A	N/A	N/A
*Therapy-response prediction*									
^51^	Prediction of response to ipilimumab in melanoma patients	No	31	31	1	N/A	N/A	70.9%	N/A
^52^	Prediction of probability that tissue from non-small-cell lung cancer will respond to immunotherapy	No	56	56	2	0.65	N/A	N/A	N/A
*Survival prediction*									
						*C* score	Hazard ratio		
^48^	Prediction of 5-year disease-specific survival in patients with colorectal cancer	No	420	420	1	N/A	2.3		AUROC = 0.96
^75^	Consensus molecular subtyping of colorectal cancer and predication of overall survival	No	769	N/A	2	0.8	N/A		N/A
^45^	Prediction of survival in colorectal cancer	Yes	1382	N/A	3	N/A	1.63 (1.14–2.33, *P* = 0.008)		N/A
^47^	Prediction of survival for patients with intrahepatic cholangiocarcinoma	No	246	246	2	N/A	0.86		N/A
^76^	Classification of patients to high risk or low risk in order to predict overall survival	No	1299	1299	2	N/A	1.74 (1.16–2.61, *P* = 0.006)		AUROC = 0.58
^77^	Stratification of patients into groups of short- and long-term survival by means of tumour- infiltrating lymphocytes	No	70	70	1	0.87	N/A		N/A
^46^	Prediction of survival in mesothelioma and identification of histological correlates	Yes	3037	3037	2	0.66	N/A		N/A
^49^	Prediction of development of metastatic recurrence in primary melanoma patients	Yes	263	263	5	N/A	N/A		AUROC = 0.91
^78^	Stratification of patients with colorectal cancer to good, uncertain or poor prognosis	Yes	4515	3595	4	N/A	3.83		Accuracy = 76%; sensitivity = 52%; specificity = 78%; PPV = 0.19; NPV = 0.94
^79^	Classification of patients with brain cancer in four groups based on survival time after diagnosis	Yes	664	454	2	N/A	N/A		AUROC = 0.96; accuracy = 80%
^80^	Prediction of overall survival of patients with hepatocellular carcinoma	Yes	732	522	2	0.7	4.3		N/A
^81^	Prediction of disease-specific survival in ten different cancer types	No	12,095	4880	10	61.1 (57.2, 65.1)	1.48 (*P* < 0.0001)		AUROC = 0.64 (58,70.3); 5-year disease- specific survival

*N/A* not available.

For each study, the level of evidence (presence of external validation), the number (#) of tissue slides, patients and patient cohorts as well as quantitative performance metrics are given, including area under the receiver-operating curve, AUROC; *F* score; accuracy; positive predictive value, PPV; negative predictive value, NPV; true-positive rate, TPR; false-positive rate, FPR; false-negative rate, FNR; other metrics (sensitivity, specificity and others) if reported in the study. This table is related to Fig. 2b.

### Prediction of genotype and gene expression

Oncogenic driver mutations change normal cells into malignant cancer cells, rewiring the cellular machinery and fundamentally changing cellular behaviour.^33,34^ Accordingly, such genetic driver mutations confer changes in the morphology of cancer cells, such as the nuclear and cytoplasmatic texture, size and shape within a histological image. Furthermore, malignant cells can also induce responses in neighbouring non-malignant cells such as fibroblasts and lymphocytes, leading to second-order morphological changes in tumour tissue on a micrometer or millimeter scale.^35^ Although each of these morphological features caused by single oncogenic driver mutations might be subtle, studies have shown that these changes can be reliably detected by DL. Indeed, merely observing these morphological patterns in H&E images allows the genotype of individual genes to be predicted directly from routine histology images. The first systematic DL-driven study in this area demonstrated how cancer genotype was reflected in the histological phenotype of lung adenocarcinoma (Table 2 and Fig. 2b): Coudray and coworkers showed that, as well as the automated detection and classification of tumours, specific genetic mutations, including those in serine/threonine kinase 11 (*STK11*), tumour protein p53 (*TP53*) and epidermal growth factor receptor (*EGFR*) could be predicted from histology alone, with AUROC values reaching up to 0.85, which they validated in an external cohort.^26^ Another study showed that the genotype of the oncogene speckle-type BTB/POZ protein (*SPOP*) could be predicted from H&E-stained images of prostate cancer, albeit with a reduced classification performance.^36^ Similarly, in melanoma, the NRAS proto-oncogene (NRAS) and B-Raf proto-oncogene (BRAF) mutational status was predictable directly from H&E images.^37^

Predicting the mutational status of these genes is relevant for targeted therapy. In lung cancer, the genotype of *EGFR* guides the use of treatment with multiple tyrosine kinase inhibitors (TKI) of the mutated *EGFR* protein, and in melanoma, mutated BRAF is directly targetable with a serine/threonine kinase inhibitor. Thus, detecting mutations in these genes directly from routine histology could have broad implications for clinical workflows. Another clinically relevant example concerns cancer immunotherapy. MSI, the genetic correlate of mismatch-repair deficiency (dMMR), is one of a few FDA-approved genetic biomarkers for the use of immune-checkpoint inhibition therapy, and the only one applicable to any type of cancer. MSI causes a strong morphological change in the tumour and its microenvironment, and can reliably be detected from histology alone in gastric, colorectal and endometrial cancer.^27^ Multiple studies have validated these findings as well as extending DL-based genotyping to a range of other mutations and gene expression markers across multiple tumour types (Table 2 and Fig. 2b). Studies published over the past 1–2 years have pursued a “pan-cancer pan-mutation” approach to try to predict any genetic alteration in any type of solid tumour directly from H&E histology.^38–40^ However, these studies have been largely based on one particular dataset, “The Cancer Genome Atlas (TCGA)”, provided by the National Cancer Institute (NCI), and so large-scale validation in genomically characterised cohorts beyond TCGA is needed to gauge the robustness of these methods in pan-cancer applications.

Currently, detecting any genetic change in tumour tissue in clinical routine requires wet-lab assays, such as IHC, in situ hybridisation (ISH), polymerase chain reaction (PCR) or next-generation sequencing (NGS), performed in parallel with the routine evaluation of histology samples, such as tumour subtyping and grading. Although these wet-lab assays vary in terms of sensitivity and specificity, they share a common set of disadvantages: they tend to be expensive and time-consuming and are not available at every point of cancer care. By contrast, DL-based evaluation of scanned routine histology slides does not incur any significant cost or time and could be deployed even on mobile hardware.^39^ Notably, however, in all DL-based studies carried out so far, the performance (as measured by AUROC) has varied according to the sample size of the training cohort and the phenotypic strength of the particular genetic target, but has been consistently inferior to the gold standard wet-lab tests (Table 1). Technological advances and training on larger datasets, however, are expected to boost performance. Furthermore, even imperfect DL-based tests could be used to prescreen patients for a genetic alteration of interest, as will be discussed below.

### Survival prediction through DL biomarkers

At almost any branch of the therapeutic decision-making tree in oncology, the risk of relapse or death must be taken into account. For example, for patients with stage II or III colorectal cancer (CRC), a high risk of relapse provides a reason to perform adjuvant chemotherapy after surgery,^41^ and for stage IV CRC, a high risk of death can prompt oncologists and patients to choose a more aggressive systemic therapy than the one currently recommended in guidelines.^42^ Currently, survival is estimated by clinical parameters such as age, gender, cancer stage, pre-existing conditions, genetic alterations and histology risk factors. These histology risk factors, which are abundant, include tumour cell differentiation, stromal abundance, lymphocyte fraction, lymphatic vessel invasion, vascular invasion, perineural invasion and necrosis in almost any type of solid tumour. In addition to these established risk factors, higher-level features carry prognostic information. For example, analysis of the spatial arrangement of lymphocytes showed that a high neutrophil-to-lymphocyte ratio is associated with unfavourable overall survival,^43^ or examination of sub-visual features such as chromatin texture can serve as a prognostic indicator in different solid tumours.^44^ DL can potentially integrate all of these visible and sub-visual features directly from image data to predict survival, as has been shown in a number of studies (Fig. 2a). Interestingly, while some studies have used manually defined prior parameters to train the DL network for survival predictors,^45^ other studies have used an unbiased approach and leave the feature selection entirely to the deep network,^46,47^ which means that no prognostic parameters, such as tissue type or cellular aspects, were manually identified or extracted during the process. Both approaches are still in need of being independently and prospectively validated in order to ultimately serve as the basis for risk-adjustment strategies in a clinical setting.

Several key studies have explored DL-based survival prediction in a number of cancer types. Bychkov et al. showed that it is possible to predict 5-year disease-specific survival of patients with CRC using H&E-stained tissue microarrays alone.^48^ Similarly, improvement of survival prediction, compared with state-of-the-art methods, was demonstrated in patients with CRC by prediction of OS through tissue classification.^45^

Courtiol et al. predicted OS in a large cohort of patients with malignant mesothelioma and visualised histological features associated with long or short survival identified by the DL network.^46^ Concurrently, disease-specific survival was estimated by DL-based prediction of the development of distant metastatic recurrence in patients with primary melanoma.^49^ This is a prime example showing that it is possible to train DL networks on clinical endpoints directly from histology. Moreover, this process could even reveal new morphological biomarkers by highlighting specific structures and regions. In the future, this reverse engineering of relevant features might even be helpful in identifying targets for the development of new therapies. However, so far, only a small number of publications have developed and discussed the clinical implications of DL-based survival prediction from routine histology (Table 2 and Fig. 2b). In particular, there are still no studies with clinical endpoints that have incorporated DL survival prediction into clinical workflows, although large prospective trials have evaluated clinical endpoints with other prognostic biomarkers such as the use of OncotypeDX in the TAILORx trial of breast cancer;^50^ this level of evidence is still missing from the DL literature.

### End-to-end response prediction directly from histology

The number of available options for targeted therapy for different types of cancer is constantly increasing. However, most of those therapies are effective in only a subset of patients and yet might still cause considerable side effects in non-responders. A prime example is cancer immunotherapy, which, although it has completely changed the therapeutic landscape for melanoma and lung cancer, can still leave approximately half of all patients with these tumour types without a meaningful response. DL might be key to the detection of structures and transformations in tumour tissue that could be used as predictive markers of a positive response to targeted therapies and therefore helps to identify responders while minimising the negative effects on non-responders.

Two potential ways of applying DL to routine histology images for the detection/identification of positive predictive markers are conceivable. First, DL can identify features, mutations, hormone- receptor status or similar molecular alterations that are already known to be targets of therapy approaches or proxies for treatment response. With DL being potentially time- and cost-saving, this approach could help to assign patients to the optimal therapy regime faster and more precisely. Alternatively, DL can be used to predict treatment response directly from a histological slide without being trained to detect specific predefined molecular biomarkers. This “end-to-end” workflow requires DL networks to be trained on large patient cohorts for which the specific type of treatment response is known. Because such image data are not easily obtained, few studies have investigated this (Table 2 and Fig. 2b). Notably, Harder and coworkers classified melanoma patients as responders and non-responders to ipilimumab,^51^ and Madabhushi et al. demonstrated a concept for the prediction of response to immunotherapy in patients with NSCLC directly from H&E- stained images.^52^ However, these studies only included small patient numbers, and it can be expected that the potential of DL to predict therapy response is not yet exhausted. Similarly to survival prediction networks, treatment response prediction might lead to the detection of new morphological markers on histology images, resulting in new therapeutic strategies.

## Implementation of DL biomarkers in clinical workflows

### DL-based mutation prediction for pre-screening or definitive testing

Clinical workflows for almost every major type of advanced cancer rely on molecular testing to tailor treatment to the molecular make-up of the tumour tissue; practical limitations, however, preclude universal testing. The application of DL-based genotyping in these workflows is twofold: DL biomarkers could be used to prescreen patients before genetic testing, or could ultimately replace current methods for definitive testing, the latter requiring a much higher test performance than achieved until now. Most proof-of-concept studies of DL for mutation prediction have reported AUROC values in the range of 0.70–0.90, which translates roughly to a specificity of 50% at a sensitivity of 90–95%. Although this performance is clearly below what is required of a definitive test, it might be useful for pre-screening patients for rare traits such as NTRK fusion, for example, narrowing down the population of potential carriers by 50% would alleviate the load of molecular testing needed. Considering the development of DL in digital pathology, technological advances can be expected to boost performance in the future. Accordingly, more and more DL biomarkers could exceed the threshold of AUROC 0.90, translating to specificities and sensitivities that are similar, or even superior, to those currently expected from molecular assays. In this case, DL concepts could be considered as definitive testing methods to detect mutations directly from histology slides.

### Moving towards clinical approval: where are we now?

Compared to its application in the field of radiology, applications of DL in histopathology have been slow to take off, but the research landscape is quickly moving from technology-driven towards clinically relevant studies, which focus more and more on problems and tasks with direct relevance for clinical decision-making and patient treatment. In parallel, more and more DL concepts are receiving approval from regulatory entities and finding their way into clinical application, for example, to detect intracranial haemorrhage on brain CT scans or to identify a pneumothorax or rib fracture on chest CT images.^53^ In the realm of histopathology image analysis, current FDA-approved procedures are limited to basic DL applications such as tumour detection and grading,^54^ but advanced image analysis methods could be expected to gain clinical approval in the next few years. However, the routine deployment of DL methods is still hampered by practical limitations: first, the broad implementation of DL histology into clinical practice would require the widespread availability of slide scanners and standardisation of file formats, which is currently far from routine practice in diagnostic pathology. Also, DL systems will have to be further improved in terms of performance to become clinically usable tools; when using DL systems for pre-screening, false- positive predictions can be mitigated by subsequent molecular testing, but false-negative predictions cannot be tolerated in a clinical setting.

In addition to being potentially useful tools for routine clinical practice in oncology, DL systems could be useful in clinical trials in two ways. By using “mutation prediction DL systems”, large patient cohorts could be inexpensively screened for a particular genetic feature. Recruiting a sufficient number of patients with a rare molecular alteration for a clinical trial is increasingly challenging, so DL-based analysis of histological H&E images could facilitate clinical trial recruitment by massively expediting and simplifying this process. In addition, DL systems could be trained to predict treatment response directly from H&E histopathology images, thereby essentially constituting a new class of companion diagnostics. As a word of caution, however, before the application of any new type of biomarker in routine clinical practice or clinical trials, legal and ethical aspects have to be considered in detail (Box 1). Future studies are needed to address these points specifically in the context of DL systems in oncology.

Box 1 Legal and ethical aspects

- A deep-learning-based biomarker has to be held to the same standard as any other biomarker: it has to be developed transparently according to the TRIPOD guidelines^82^ (https://www.equator-network.org/reporting-guidelines/tripod-statement/) and it should be validated in multicentric retrospective and prospective studies.
- To be used in clinical routine, a deep-learning system has to fulfil medical device standards set by the Food and Drug Administration (FDA), the European Medicines Agency (EMA) or a similar institution in other regions.
- Dynamically evolving biomarkers are a challenge for the current regulatory system. In principle, a deep-learning system can “learn on the job” and be iteratively defined. This is different than for any established prognostic or predictive biomarker, which does not change over time.

### Moving towards end-to-end systems

Genetic biomarkers in solid tumours are rarely an end in themselves, rather, they can be used as a surrogate to predict the response to a particular treatment. In the best-case scenario, the surrogate genetic marker is mechanistically related to a particular treatment and yields a high positive predictive value for treatment response. For example, mutations in, or overexpression of, *HER2* in breast cancer are predictive of a positive response to trastuzumab.^4^ However, the situation for many other molecular biomarkers is not as clear-cut. For example, the overexpression of PD-L1 in tumour tissue does not have a perfect positive predictive value for the response to anti-PD1/PD-L1 treatment in lung cancer.^55^ Consequently, end-to-end DL systems have been proposed as an alternative approach, aiming to predict the response to treatment directly from images. Response to cancer treatment is often assessed through the “Response evaluation criteria in solid tumours” (RECIST), and these criteria have been used to directly train DL networks. In these cases, RECIST status is the ground-truth label to be predicted from images. More generally, prognostic end-to-end DL systems predict survival for individual patients based on histology images without focusing on a specific type of treatment. Such end-to-end systems could theoretically outperform molecular prognostic or predictive biomarkers, as they would have the potential to predict outcome directly from a histological image without focussing on a predefined predictive parameter.

Unfortunately, patient cohorts needed for predictive end-to-end DL systems are currently unattainable to most researchers. Collaboration between clinicians, pathologists and DL researchers is key to the development of such systems in the future.

## Outlook

Within less than 2 years of the first publication on DL-based genetic testing, the application of advanced DL in histopathology has grown exponentially, promising clinical impact on a broad range of scenarios. This paper provides an overview and a quantitative comparison of different applications of this technology. Of note, quality standards in clinically applied DL histopathology are still evolving. As shown in Tables 1 and 2, there is a marked discrepancy in terms of external validation and the reporting of statistical measures between different studies. To move DL methods to clinical application, external validation should be a cornerstone of future studies. Also, transparent reporting of the number of patients, slides and cohorts included in an analysis as well as disclosure of a range of statistical measures should become the standard in the field.

Most DL classifiers still require an increase in performance to achieve the reliability that is needed for application in clinical workflows as definitive testing tools. Such improvements can be expected to be brought about by three key drivers: the availability of larger datasets with clinical annotations^23^ and improvements in both hardware and algorithms. This new class of biomarkers has the potential to change clinical workflows in oncology in the next few years, but large-scale multicentre trials are needed to verify whether this approach can live up to these hopes.

## Supplementary information

Suppl. Material

## Data Availability

Not applicable.
