# Supplementary material for: Deep learning in cancer pathology: a new generation of clinical biomarkers
Source: Br J Cancer. 2020 Nov 18;124(4):686–96. doi: 10.1038/s41416-020-01122-x (PMC7884739; doi:10.1038/s41416-020-01122-x)
Supplement: Supplementary file 1 — Suppl. Material [file 41416_2020_1122_MOESM1_ESM.docx]

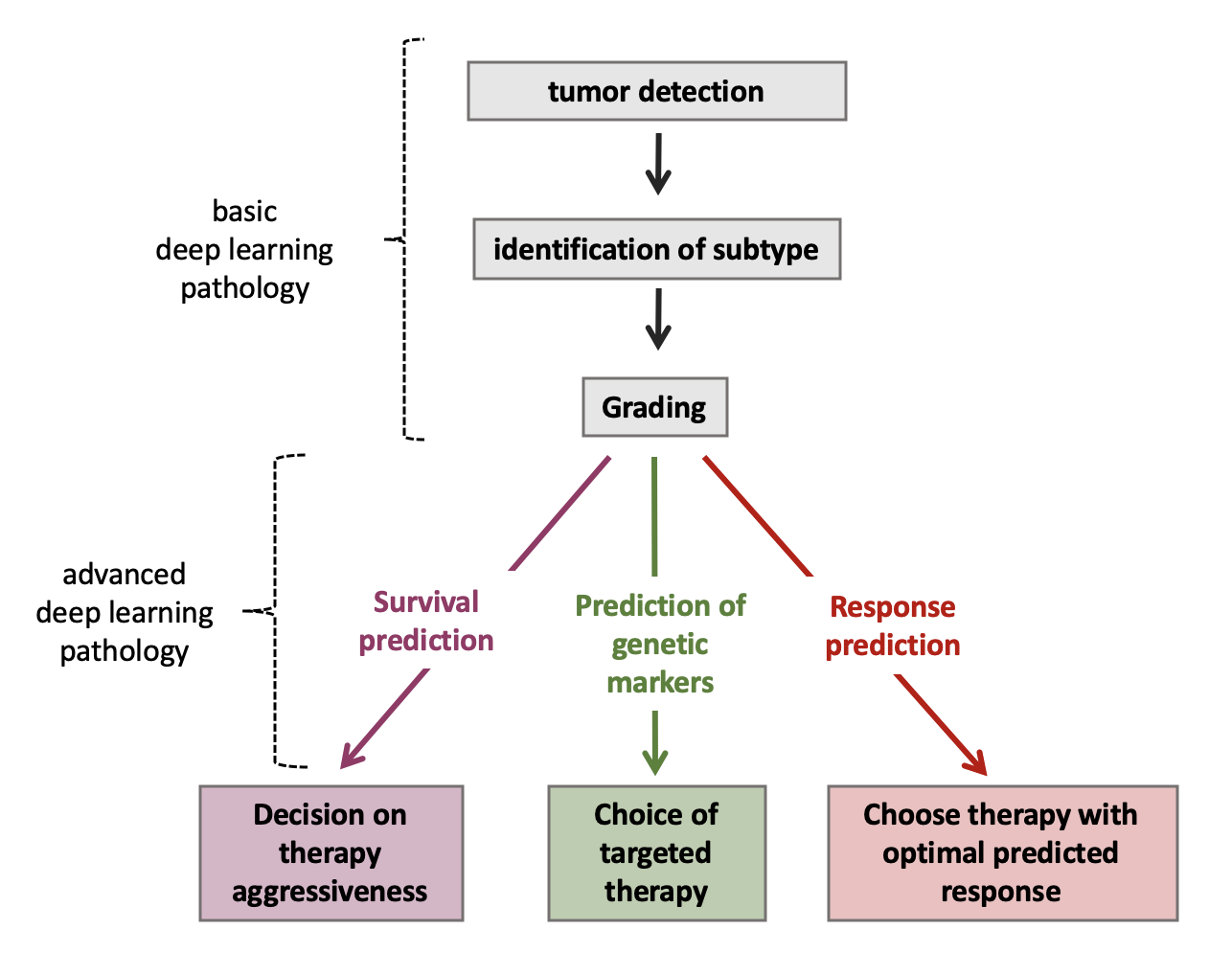
**Supplementary Figure 1: Conceptual classification of deep learning approaches in digital pathology.**


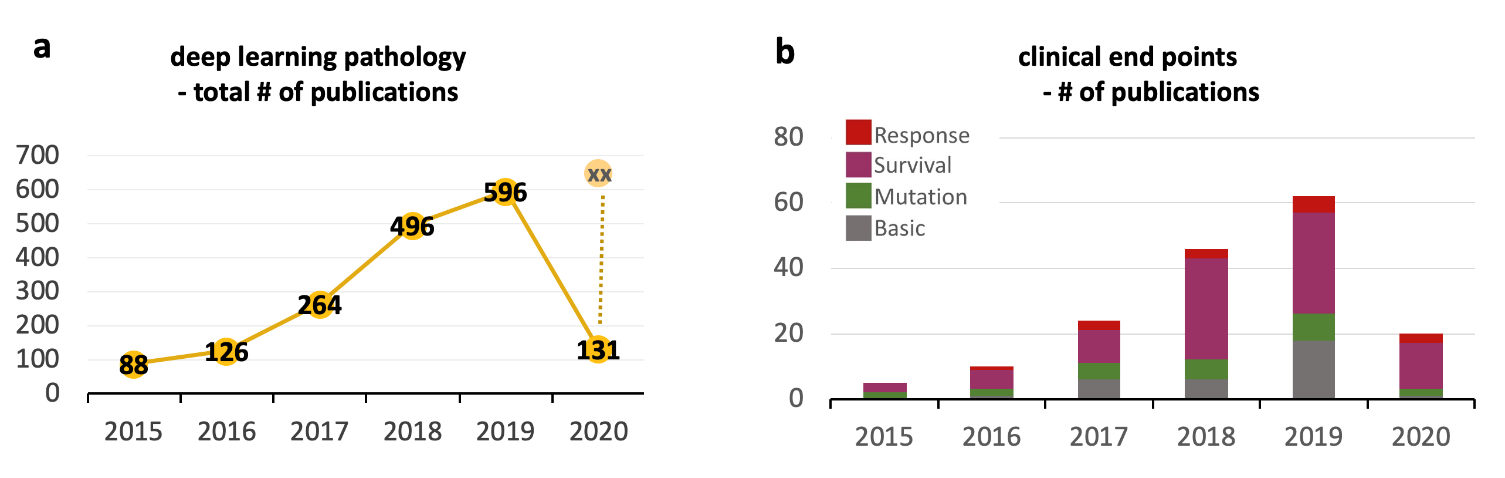
**Supplementary Figure 2**: (a) Total number of papers associated with deep learning and pathology. For details, see Suppl. Methods. (b) Number of publications for basic and advanced digital pathology approaches. Categories corresponding to Figure 2. For details, see Suppl. Methods.

# Supplementary Methods

## Literature search (related to Table 1, Table 2, Figure 2, Suppl. Figure 2)

The literature reviewed in this paper (Table 1 + Table 2) was gathered by systematic research on the Pubmed database (<https://www.ncbi.nlm.nih.gov/pubmed/>) for indexed publications as well as on the preprint server bioRxiv (<https://www.biorxiv.org/>). Additional items of high relevance were added by consensus of the authors.

In Figure 2c and Suppl. Figure 2, the number of publications were visualised by assessing the quantity of papers indexed on Pubmed by different search queries.

For Figure 2b the Pubmed search query was: (((((((((((deep learning histology) OR deep learning pathology) OR Convolutional Neural Networks pathology) OR Convolutional Neural Networks histology) OR Recurrent Neural Networks pathology) OR Recurrent Neural Networks histology) OR CNN pathology) OR CNN histology) OR RNN pathology) OR RNN histology) OR LSTM pathology) OR LSTM histology

To get quantitative estimates for the number of publications in any of four thematic categories (shown in Suppl. Figure 2), the following search queries were used:

Basic: (((((((((((deep learning histology cancer detection[Title/Abstract]) OR deep learning pathology cancer detection[Title/Abstract]) OR CNN histology cancer detection[Title/Abstract]) OR CNN pathology cancer detection[Title/Abstract]) OR convolutional neural network histology cancer detection[Title/Abstract]) OR convolutional neural network pathology cancer detection[Title/Abstract]) OR recurrent neural network histology cancer detection[Title/Abstract]) OR recurrent neural network pathology cancer detection[Title/Abstract]) OR RNN pathology cancer detection[Title/Abstract]) OR RNN histology cancer detection[Title/Abstract]) OR LSTM histology cancer detection[Title/Abstract]) OR LSTM pathology cancer detection[Title/Abstract]

Response: (((((((((((deep learning histology treatment response[Title/Abstract]) OR deep learning pathology treatment response[Title/Abstract]) OR CNN histology treatment response[Title/Abstract]) OR CNN pathology treatment response[Title/Abstract]) OR convolutional neural network histology treatment response[Title/Abstract]) OR convolutional neural network pathology treatment response[Title/Abstract]) OR recurrent neural network histology treatment response[Title/Abstract]) OR recurrent neural network pathology treatment response[Title/Abstract]) OR LSTM histology treatment response[Title/Abstract]) OR LSTM pathology treatment response[Title/Abstract]) OR RNN pathology treatment response[Title/Abstract]) OR RNN histology treatment response[Title/Abstract]

Survival: (((((((((((deep learning histology survival[Title/Abstract]) OR deep learning pathology survival[Title/Abstract]) OR CNN histology survival[Title/Abstract]) OR CNN pathology survival[Title/Abstract]) OR convolutional neural network histology survival[Title/Abstract]) OR convolutional neural network pathology survival[Title/Abstract]) OR recurrent neural network histology survival[Title/Abstract]) OR recurrent neural network pathology survival[Title/Abstract]) OR LSTM histology survival[Title/Abstract]) OR LSTM pathology survival[Title/Abstract]) OR RNN histology survival[Title/Abstract]) OR RNN pathology survival[Title/Abstract]

Mutation: (((((((((((deep learning histology mutation[Title/Abstract]) OR deep learning pathology mutation[Title/Abstract]) OR CNN histology mutation[Title/Abstract]) OR CNN pathology mutation[Title/Abstract]) OR convolutional neural network histology mutation[Title/Abstract]) OR convolutional neural network pathology mutation[Title/Abstract]) OR recurrent neural network histology mutation[Title/Abstract]) OR recurrent neural network pathology mutation[Title/Abstract]) OR RNN histology mutation[Title/Abstract]) OR RNN pathology mutation[Title/Abstract]) OR LSTM histology mutation[Title/Abstract]) OR LSTM pathology mutation[Title/Abstract]

All literature items resulting from the above described search were manually screened for relevance. Articles were deemed relevant if they were related to histological images analyzed by deep learning methods. Only articles published before 27 March 2020 were considered for this review.
